# Hybrid deep learning model based smart IOT based monitoring system for Covid-19

**DOI:** 10.1016/j.heliyon.2023.e21150

**Published:** 2023-10-18

**Authors:** Liping Yu, M.M. Vijay, J. Sunil, V.G. Anisha Gnana Vincy, Vediyappan Govindan, M. Ijaz Khan, Shahid Ali, Nissren Tamam, Barno Sayfutdinovna Abdullaeva

**Affiliations:** a School of Computer Science and Technology, Shandong Technology and Business University, Yantai, 264005, China; b SCAD College of Engineering and Technology, Tirunelveli, India; c Department of Computer Science and Engineering, Annai Vailankanni College of Engineering, Kanyakumari, India; d Department of Computer Science and Engineering, Danish Ahmed College of Engineering, India; e Department of Mathematics, Hindustan Institute of Technology and Science (Deemed to be University), Padur, Kelambakkam, 603103, India; f Department of Mechanical Engineering, Lebanese American University, Kraytem, Beirut, 1102-2801, Lebanon; g School of Electronics Engineering Peking University, Beijing, China; h Department of Physics, College of Science, Princess Nourah bint Abdulrahman University, P.O. Box 84428, Riyadh, 11671, Saudi Arabia; i Pedagogical Sciences, Vice-Rector for Scientific Affairs, Tashkent State Pedagogical University, Tashkent, Uzbekistan; j Department of Mathematics and Statistics, Riphah International University I-14, Islamabad 44000, Pakistan

**Keywords:** Healthcare monitoring system, Quarantine practices, Puzzle optimization algorithm, IoT monitoring system, Oxygen level

## Abstract

Recently, COVID-19 becomes a hot topic and explicitly made people follow social distancing and quarantine practices all over the world. Meanwhile, it is arduous to visit medical professionals intermittently by the patients for fear of spreading the disease. This IoT-based healthcare monitoring system is utilized by many professionals, can be accessed remotely, and provides treatment accordingly. In context with this, we designed an IoT-based healthcare monitoring system that sophisticatedly measures and monitors the parameters of patients such as oxygen level, blood pressure, temperature, and heart rate. This system can be widely used in rural areas that are linked to the nearest city hospitals to monitor the patients. The collected data from the monitoring system are stored in the cloud-based data storage and for the classification our approach proposes an innovative Recurrent Convolutional Neural Network (RCNN) based Puzzle optimization algorithm (PO). Based on the outcome further treatments are made with the assistance of physicians. Experimental analyses are made and analyzed the performance with state-of-art works. The availability of more data storage capacity in the cloud can make physicians access the previous data effortlessly.

## 1 Introduction

Medical amenities serve as a fantastic illustration of healthcare, diverse categories of patients with diverse requirements are provided with an extensive variety of specializations and resources [1]. Insufficient space is one of the issues facing hospitals today; this issue arises when the quantity of individuals receiving treatment exceeds the capacity of the hospital's treatment centers, healthcare workers, or infrastructure. Patients must wait longer in hospitals when they are congested, while medical personnel's standard of treatment and productivity suffer, overpopulation is a worldwide challenge [2] and an illness that impacts everyone, regardless of status in society. This issue's recurrence is described to several elements, particularly client input and discharge, lengthy wait times for patients, medical practitioners' effectiveness, agglomerations, and the accessibility of facilities. Population growth [3] causes a decline in the standard of care, a boost in mortality rates [4], and a rise in social prejudice among those waiting in lines. All of the situations mentioned earlier have been seen to grow in frequency with the rapidity at which COVID-19 illness is expanding. Consequently, the rising prevalence of COVID-19 creates a new problem for the healthcare [5] industry along with the daily services needed to be delivered, where an elevated likelihood of death is anticipated and a lengthy assessment procedure.

Globally, the COVID-19 coronavirus outbreak is driving a rise in the popularity of using face shields in general. People utilized to use covers to safeguard themselves from contaminants in the air before COVID-19. People who are embarrassed about the way they appear also conceal how they are feeling from others by covering their actual faces. Investigators have demonstrated that using a face mask prevents the spread of COVID-19, it is the most recent pandemic virus to affect humanity in the past decades. The World Health Organization [6] had to designate COVID-19 as a worldwide epidemic in 2020 due to its swift propagation, claiming that in less than six months, COVID-19 affected more than 5 million individuals in 188 nations around the globe. Intimate touch, congested, and congested environments are all conducive to the virus's propagation. It's crucial to find COVID-19 patients immediately to segregate them and stop the illness from spreading across the population. On the other hand, given COVID-19's current pattern of disseminating proper identification continues a difficult endeavor, especially in areas with few healthcare facilities. Presently, the only way the globe can combat this coronavirus is to stop it from spreading by taking precautions like masks for the face, washing your hands, and isolation from society. Using early detection of additional instances and tracking their progression, innovation can nonetheless also aid in slowing the epidemic's progression [7]. Buses serve as both a regular mode of communication and a gathering venue for everyone in the community throughout Covid-19.

On account of the volume of people, several health reasons precautions have been implemented, namely scheduled sterilization and an interpersonal buffer between commuters. In addition, drivers of buses are more inclined to connect with clients in particular ways. It is anticipated to be able to identify mask wearers to facilitate activities like crowd surveillance and noncontact climate assessment during pandemics and other scenarios. Global air pollution [8] has significantly decreased as a result of the COVID-19 shutdown, especially in large industrialized nations. This is a significant and instant good effect.

Excessive fluctuations in mood, anger, departure, and disturbances in emotion can all be signs of depressive disorders. It's typical to have physical manifestations like exhaustion, and migraines, as well as others that have no known clinical cause, such as those caused by binge eating and harming oneself. For these reasons the remote monitoring of COVID-19 patients is essential and for that, we propose a novel approach that uses smart monitoring system, and the data collected from the system are classified using the innovative POA-based RCNN approach. Major contributions are.

➢ The proposed work designed a smart remote monitoring system for COVID-19 patients that utilize effective sensors. This helps in observing data accurately and at the same time, the power consumption and effectiveness are escalated.
➢ The data observed from the sensors are processed using the proposed POA-RCNN approach which effectively classifies the data as COVID-19 and normal.

The remainder of the work is arranged as; the state-of-art works are analyzed with their effectiveness and demerits in section 2. The proposed methodology is stated in section 3. The results and discussion section are presented in section 4. Finally, the work is concluded in section 5.

## 2 Literature survey

Abdul Kareem et al. [9] have described Machine Learning (ML) to identify COVID-19 cases in modern facilities. A new class of healthcare called smart hospitals is based on suggested technologies and constructed using various distinct service infrastructures. These medical facilities frequently have two innovations that are frequently used in particular healthcare specialties. Intelligent healthcare facilities are considered unique because it combines several tasks, including administration, making choices, assessment, and therapy. It is useful for diagnosing COVID-19 instances, which lowers computational burden and evaluation period. Hence, the accessibility and affordability of imaging is increased. Loey et al. [10] have presented a hybrid model using deep and classical machine learning that will be demonstrated for mask-based identification. The suggested methodology enables the identification of individuals without using face coverings, which may be coupled with safety cameras to prevent Covid-19 spreading. The situations in which face masks were worn into three distinct classifications. Appropriate face covering possessing, wrong face mask consumption, and no face shield usage are the three categories. The face identification stage of the suggested technique had a 98.70 % efficiency rate. Therefore, it fails to provide the best precision and the least amount of time spent.

Sakib et al. [11] have evaluated a deep learning-based chest radiograph classification (DL-CRC) structure to accurately identify COVID-19 instances as opposed to irregular and typical situations. Imaging tests from fit and people suffering from pneumonia documented to this point are combined into a singular database from several open-access venues. The primary cause is determined to be the information set's tiny proportion of COVID-19 occurrences. Using our suggested approach greatly raised the rate of categorization performance to 94.61 %. Thus, the processing time is reduced. Awais et al. [12] have implemented the Internet of Things (IoT) enabled platform for psychological assessment. The tool was employed to identify the intensity and excitement feelings brought on by rhythmic lyrics. According to this, video footage concerning feelings was used to categorize them by linking gestures with electrocardiogram breathing patterns. It offers smooth data transfer among the devices that detect the gateway with minimal delays and excellent dependability. Hence, increased edge service viability cannot be achieved with current methods. Otoom et al. [13] have demonstrated an Internet of Things (IoTs) to track the medical responses of people who have previously benefited from the infection, to promptly recognize probable coronavirus infections, and to comprehend the characteristics of the disease by gathering and analyzing pertinent information from patients. Utilizing actual time illness facts will allow reliable and efficient detection and classification of prospective COVID-19 patients [[14], [15], [16], [17], [18]]. This approach would enable monitor of cases that were treated and a deeper comprehension of the illness. Nevertheless, the complicated nature of processing information becomes a barrier.

Kong et al. [14] suggested an edge computing-based mask identification framework (ECMask) help in the protection of the community's health, which can guarantee the continuous operation of the buses' inexpensive cameras. The facial expression classifier is then developed and verified using our bus movement tracking database as well as datasets that are freely accessible. After acquiring and emerging the facial regions of the Bus Drive Tracking Dataset, an opaque mask verification simulation can be received training. The reliability of identifying faces can then be increased following potential video recovery [[18], [19], [20], [21], [22]]. Thus, it uses a lack of connectivity to broadband to prevent information from accessing its facilities. Wang et al. [15] highlighted hybrid machine-learning techniques in the two phases to identify mask wearers. Predetection and testing are the two steps that it is intended to have. The Faster_RCNN platform uses an indirect learning method to achieve detection. A multiple-class recognition system serves as the foundation for the refined identification method. Such inaccuracies can be successfully addressed and verified. Therefore, the face area that is nearly completely covered by a mask, and clinical glasses provides complications for our approach [[22], [23], [24], [25]]. Barnawi et al. [16] have developed the Internet of Things Unmanned aerial vehicles (IoTUAV) that depends on the way to get unprocessed data using inbuilt temperature sensors. The technique now includes a system for input that checks to see if the individual who was reported to have an elevated temperature turns out to have COVID-19 after getting checked out at the hospital. The captured recordings may subsequently be used to alert neighbors and request separation and examination from those who are close to the sick there [[17], [18], [19], [20]]. Hence, making it feasible to collect data without merging is a big issue that has to be solved.

## 3 Methodology

**Fig. 1:**
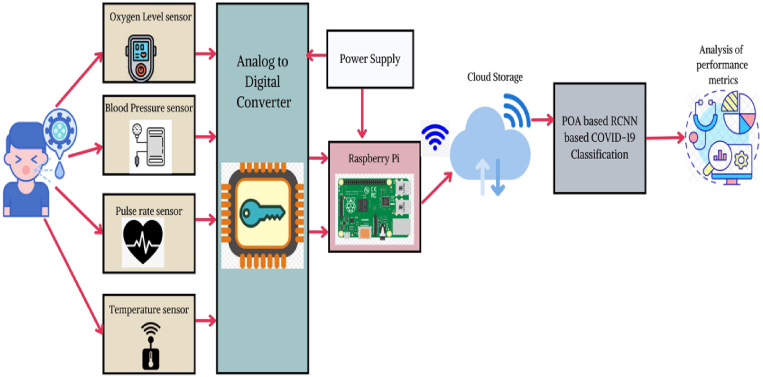
Overlay of proposed IoT-based Smart monitoring system for COVID-19.

The proposed approach is based on IoT based smart monitoring system for COVID-19 patients and it includes several hardware elements that are mainly required for the designing of the prototype [[25], [26], [27], [28], [29]]. Some of the hardware components are a contactless temperature sensor, a microcontroller with an inbuilt analog-to-digital converter, an oximeter, and a blood pressure sensor. For COVID-19 patients it is necessary to monitor the oxygen level, temperature, and blood pressure to keep the patients safe. All the sensors used are interfaced with the Raspberry Pi microcontroller to save the information in the cloud storage. Henceforth, the stored data are analyzed and classified using the proposed POA-based RCNN approach which efficiently classifies COVID-19 as normal. The overall framework of the proposed approach is depicted in Fig. 1.

For the non-contact temperature measurement, we have adopted the MLX90614 sensor which is combined with a less noise amplifier. It achieves higher resolution accuracy with the 17-bit ADC and DSP unit which is powerful. It is a small-size single-board computer that can be run in Android and Linux versions and is designed to work over the ARM processor. It usually includes sensors and external devices with inbuilt displays along with the LAN port for further communication. Meanwhile, it can be programmed using the PYTHON and that's the reason it is explicitly used in many applications. Liang et al. in their study [28], investigated the impact of device-free motion using RFID for trajectory detection. Zeng et al. [29] focused on the advancement of NMR signals in metal-organic systems trapped in aqueous solutions. Li et al. [30] proposed a multi-agent based supply chain framework to handle uncertain demands and switching topology. Li et al. [31] conducted research on the 2019 coronavirus epidemic among teachers in China. Jian et al. [32] proposed a small heterogeneous cell networking approach for energy-efficient IoT networks. Song et al. [33] developed a method for identifying performance anomalies in fluctuating cloud environments. Lu et al. [34] presented an adaptive control framework for time delay tele-operation with uncertain dynamics. The pulse oximeter used here is MAX30100 which is also used to monitor the heart rate. It consists of a photodetector, a low-noise analog signal processing unit, two LEDs, and optimized optics. This is usually wrapped around the arm and provides three types of signals to the microcontroller. The first value is systolic, the second diastolic, and the last one is the pulse rate. In our work, we have adopted the sensor BP001 with a pressure range of 0–300 mg. Moreover, the accuracy is accurate. Our proposed approach follows three steps namely, (i) Data capturing, (ii) Data processing using the proposed POA-RCNN approach, and (iii) Performance metrics [[35], [36], [37], [38], [39]]. Liao et al. [40], Cheng et al. [41], and Zhuang et al. [42] respectively worked on integrated multi-task models for fake news detection, dynamic service coordination in IoT environments, and large lung CT image databases using WSSENet-based similarity retrieval techniques. In another study, Zhuang et al. [43] applied the same technique to mobile telemedicine networks. References [[44], [45], [46]] highlight the applications of medical technologies and IoT.

### 3.1 Data capturing

This is the initial step and is used for the collection of data from the sensors as mentioned in Fig. 1. The sensors are connected to the Raspberry Pi microcontroller with the GPIO pins and the output pins of sensors are connected to the RX pin of the Raspberry Pi. A power supply of +5 V is applied to the microcontroller and the sensors. Here we used an SMPS power supply. The sensors are purely IC based and hence the utilization of load current is low. However, the utilization of SMPS also reduced efficiency due to heat dissipation. This system can be used to monitor 5 different patients simultaneously with higher efficiency.

### 3.2 Data processing using the POA-RCNN approach

The captured data are processed with the assistance of the proposed approach. This approach escalates the classification accuracy of data as COVID-19 and normal [20]. This would explicitly help the physician to do effective treatment instantly without any fatality. The details are elucidated in the following section. The simulation of a puzzle game develops the population-oriented puzzle optimization algorithm (POA). The population of the best member is selected and the other member population takes the guidance to solve each puzzle in POA [17]. The problem variable values are identified using every population member and the mathematical outlines of POA is shown as follows;

(1) $$Y={\left[\begin{array}{l} Y_{1}\\ \vdots \\ Y_{j}\\ \vdots \\ Y_{M} \end{array}\right]}_{M\times n}={\left[\begin{array}{l} Y_{1}⥄⥄⥄⥄⥄\cdots ⥄⥄⥄⥄⥄⥄⥄Y_{1,D}⥄⥄⥄⥄⥄⥄⥄\cdots ⥄⥄⥄⥄⥄⥄⥄Y_{1,n}⥄\\ \vdots ⥄⥄⥄⥄⥄⥄⥄⥄⥄⥄\ddots ⥄⥄⥄⥄⥄⥄⥄⥄\vdots ⥄⥄⥄⥄⥄⥄⥄⥄⥄⥄⥄⥄⋰⥄⥄⥄⥄⥄⥄⥄⥄⥄⥄\vdots ⥄\\ Y_{j,1}⥄⥄⥄⥄⥄\cdots ⥄⥄⥄⥄⥄⥄⥄Y_{j,D}⥄⥄⥄⥄⥄⥄⥄⥄\cdots ⥄⥄⥄⥄⥄⥄⥄Y_{j,n}⥄\\ ⥄⥄\vdots ⥄⥄⥄⥄⥄⥄⥄⥄⋰⥄⥄⥄⥄⥄⥄⥄⥄\vdots ⥄⥄⥄⥄⥄⥄⥄⥄⥄⥄⥄⥄\ddots ⥄⥄⥄⥄⥄⥄⥄⥄⥄⥄\vdots ⥄\\ Y_{M,1}⥄⥄⥄⥄\cdots ⥄⥄⥄⥄Y_{N,D}⥄⥄⥄⥄⥄⥄\cdots ⥄⥄⥄⥄⥄⥄Y_{M,n}⥄ \end{array}\right]}_{M\times n}$$

The *j*th puzzle with the population is $Y_{j}$ and the number of population is *M* and the problem variables are *n*. The search space dimension is D. The objective function receives a similar amount of member population.

(2) $$F={\left[\begin{array}{l} fun_{1}\\ \vdots \\ fun_{j}\\ \vdots \\ fun_{M} \end{array}\right]}_{M\times 1}={\left[\begin{array}{l} F\left(Y_{1}\right)\\ \vdots \\ F\left(Y_{j}\right)\\ \vdots \\ F\left(Y_{M}\right) \end{array}\right]}_{M\times n}$$

Based on the objective function (fun), the vector value is *F*. Determine the best member (*best*) as,

(3) Best=Yi,⥄fun=iMin(F)

Where, $fun_{i}$ and $Y_{i}$ represents the minimal objective function and *i*th puzzle. Depending upon the control of other members, update the population of every member ($Y_{j}^{new}$). Depending upon the usage of puzzle pieces, the population of every member attempt to finish it puzzle.

(4) $$Y_{j}^{new}=Y_{i}+Random\times DY_{j}$$

(5) $$Y_{j}=\left\{ \begin{array}{l} Y_{j}^{new},⥄⥄⥄⥄⥄⥄⥄⥄⥄⥄⥄⥄⥄⥄F_{j}^{new}<F_{j}\\ Y_{j},⥄⥄⥄⥄⥄⥄⥄⥄⥄⥄⥄⥄⥄⥄⥄⥄⥄⥄Otherwise \end{array}\right.$$

The number of recommended puzzle pieces is $M_{r}$. The current and maximum number of iterations are depicted using *t* and *T*.

(6) $$N_{r}=Round\left(0.5\times \left(1-\frac{t}{T}\right)\times M\right)$$

Determine the new population member status depending upon the iterations. To solve optimization issues, POA accomplishes optimal quasi-best solutions after finishing iterations. The structure of a faster RCNN has the major role of a regional proposal network (RPN). The whole network detection accuracy and computational efficiency are increased with the help of POA because it is easy to implement, flexible, has minimum time consumption, has better ability of convergence, etc. For these reasons, we adopt POA with RCNN to categorize Covid 19 patients [18]. Depending upon the mechanism of a sliding window, the candidate regions were created with the help of RPN. 256-dimensional Eigenvector obtained using the convolution feature layer via the sliding window. At every position, obtain *k* anchor boxes, center point, and convolutional feature map. The coordinates of the central position (y, z) and candidate region present and evaluate the full connection score. The whole network output performance is affected by the anchor boxes’ width, length, and size. These parameters are tuned using POA. The bounding box detection loss $Dec_{loss}$ and classification loss compose a loss function of RPN.

(7) $$Loss\left(P_{j},T_{j}\right)=\frac{1}{M_{Cla}}\sum_{j}Cla_{loss}\left(P_{j},P_{j}^{*}\right)+\frac{1}{M_{Dec}}\sum_{j}T_{J}^{*}Dec_{loss}\left(T_{j},T_{j}^{*}\right)$$

(8) $$Cla_{loss}\left(P_{j},P_{j}^{*}\right)=-\log\left[\left(P_{j},P_{j}^{*}\right)+\left(1-P_{j}\right)\left(1-P_{j}^{*}\right)\right]$$

(9) $$Dec_{loss}\left(T_{j},T_{j}^{*}\right)=\sum_{J}Smooth_{loss_{1}}\left(T_{j},T_{j}^{*}\right)$$

(10) $$Smooth_{loss_{1}}\left(y\right)=\left\{ \begin{array}{l} 0.5y^{2},⥄⥄⥄⥄⥄⥄⥄⥄⥄⥄⥄⥄\left|y\right|<1\\ \left|y\right|-0.5,⥄⥄⥄⥄⥄⥄⥄⥄\left|y\right|\geq 1 \end{array}\right.$$

The entire amount of anchor coordinates concerning COVID-19 are $M_{Dec}$ and $M_{Cla}$ with the probability is $P_{j}$ and sample label is $P_{j}^{*}$. Both real and predicted coordinates of bounding boxes $\left(T_{j}⥄and⥄⥄T_{j}^{*}\right)$. Based on the reasonable time, POA tunes the RCNN and demonstrates the maximal classification outputs. Without trap local optima, each puzzle search space applies the best solution to fine-tune the RCNN. Update equation (5) to achieve the optimal best result for RCNN fine-tuning [19]. Equation (11) updates the fitness function for evaluating the error of classification.

(11) $$F=\frac{Number⥄of⥄misclassified⥄⥄patients}{Total⥄⥄number⥄⥄of⥄⥄patients}\times 100\%$$

**Fig. 2:**
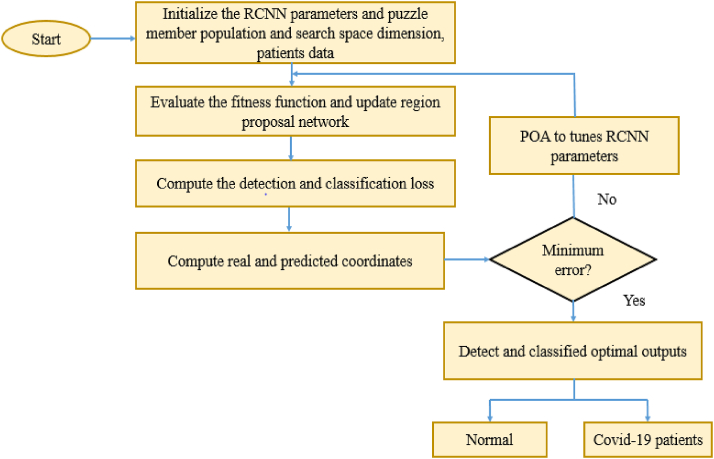
Flowchart of POA-based RCNN for covid-19 patient classification.

POA with the RCNN model accomplishes maximal classification accuracy with minimum error rate thereby classifying both normal and covid-19 patients. A flowchart based on covid-19 patient classification via POA-based RCNN is outlined in Fig. 2.

### 3.3 Performance metrics

The performances of the proposed system are analyzed to study the effectiveness of the proposed work. For this purpose, we have utilized the performance metrics such as accuracy, sensitivity, and specificity [38,39,47,48]. Accuracy determines how accurately the COVID-19 patients’ data are evaluated with the assistance of the proposed system. It is interpreted as,

$$A=\frac{\left(TP1+TN1\right)}{TP1+FP1+FN1+TN1}$$

Sensitivity determines the probability of how our system works over the dataset to detect the patients. It is interpreted as,

$$SN=\frac{TP1}{TP1+FN1}$$

Specificity determines the probability that how efficiently our approach detects the normal dataset. It is interpreted as follows,

$$SP=\frac{TN1}{TN1+FP1}$$

here, TN1 indicates the True negative values and TP1 implies the true positive value with the false negative and positive values as FP1 and FN1 respectively.

## 4 Results and discussion

The proposed system is tested over various patients to analyze the performance and for that parameter such as SPO2, body temperature, heart rate, and blood pressure are taken. The comparative analysis based on the proposed system and commercial sensors is made for further analysis. The comparative statistical analysis based on the body temperature measurement of the proposed and commercial sensor is outlined in Table 1. Here, the relative errors are ranges from 0 to 2.82. In the table, we have listed the actual room temperature, body temperature, and measured body temperature of the patients. The sensor used in our work is MLX90614.

**Table 1:** Comparative study based on proposed MLX90614 and commercial sensors.

Samples	Measured room temperature	Body temperature	Measured body temperature	Error (%)
**1**	28 °C	34 °C	35 °C	2.9
**2**	29 °C	35 °C	35 °C	0
**3**	30 °C	35 °C	36 °C	2.82

The comparative study based on the proposed sensor and commercial sensor for heart rate measurement is illustrated in Table 2. Here we have taken 3 samples and analyzed the performance. The error rate of the proposed sensor ranges from 0 to 2.84 %.

**Table 2:** Comparative study based on Heart rate sensor and commercial sensors.

Samples	Bpm present	Measured bpm	Error (%)
**1**	70	70	0
**2**	69	71	2.84
**3**	72	70	2.74

The comparative study based on the proposed oximeter and commercial oximeter is framed in Table 3. For that, we measured the SPO_2_ and actual SPO_2_. The error rate is evaluated and ranges from 0 to 1.07 as shown in Table 3.

**Table 3:** Comparative study based on oximeter sensor and commercial sensors.

Samples	SPO_2_ present	Measured SPO_2_	Error (%)
**1**	98	99	1.03
**2**	99	99	0
**3**	95	96	1.07

**Fig. 3:**
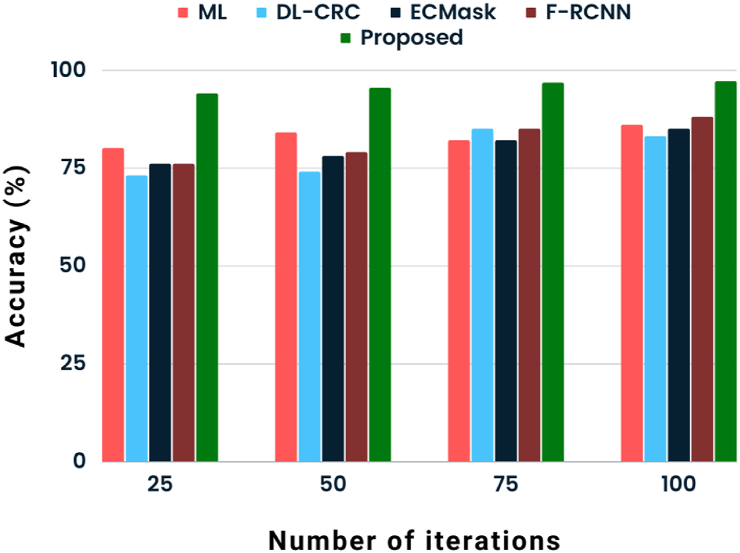
State-of-art analysis of accuracy.

The collected data using the blood pressure sensor is listed in Table 4. Here we have measured both diastolic and systolic measurements of three samples and listed. The output of the proposed classification approach and state-of-art works such as ML [9], DL-CRC [11], ECMask [14], and F-RCNN [15]. For the comparison, we have taken the parameter metrics mentioned previously. The graphical representation of accuracy rate analysis about various methodologies is shown in Fig. 3. To demonstrate how well the proposed POA-RCNN classifier outperforms with ML [9], DL-CRC [11], ECMask [14], and F-RCNN [15] approaches, the accuracy rate it achieves is compared to that of existing methods. There were 25–100 iterations in total. The proposed POA-RCNN classifier's accuracy rate at the 100th iteration is 97.12 %, compared to other compared methods' accuracy rates of 86 %, 83 %, 85 %, and 88 % for ML, DL-CRC, ECMask, and F-RCNN methods, respectively.

**Table 4:** Proposed system based collected blood pressure data.

Samples	Diastolic	Systolic
**1**	66	114
**2**	90	140
**3**	65	100

**Fig. 4:**
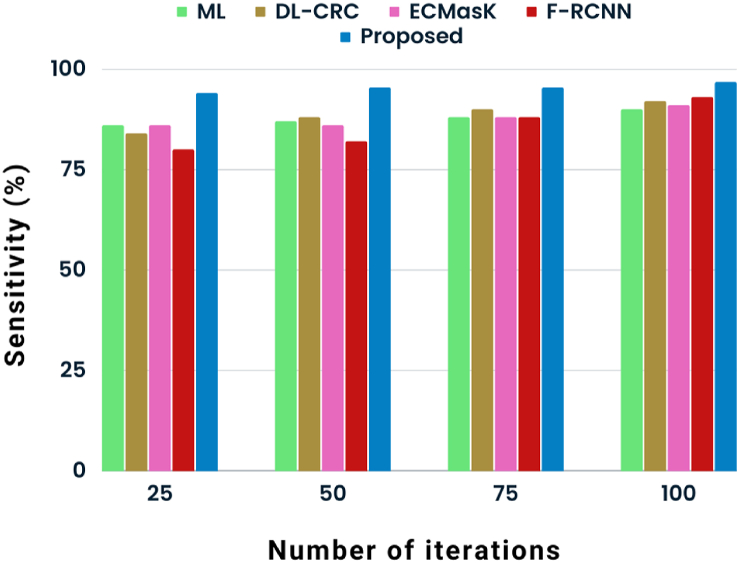
State-of-art analysis of sensitivity.

Fig. 4 illustrates the graphical representation of sensitivity rate analysis concerning several approaches. The sensitivity rate that the proposed POA-RCNN classifier obtains is compared to that of existing techniques to show how well it outperforms ML [9], DL-CRC [11], ECMask [14], and F-RCNN [15] approaches. Overall, there were 25–100 iterations. The sensitivity rate of the proposed POA-RCNN classifier at the 100th iteration is 96.74 %, while the sensitivity rates of the ML, DL-CRC, ECMask, and F-RCNN methods, respectively, are 90 %, 92 %, 91 %, and 93 %.

**Fig. 5:**
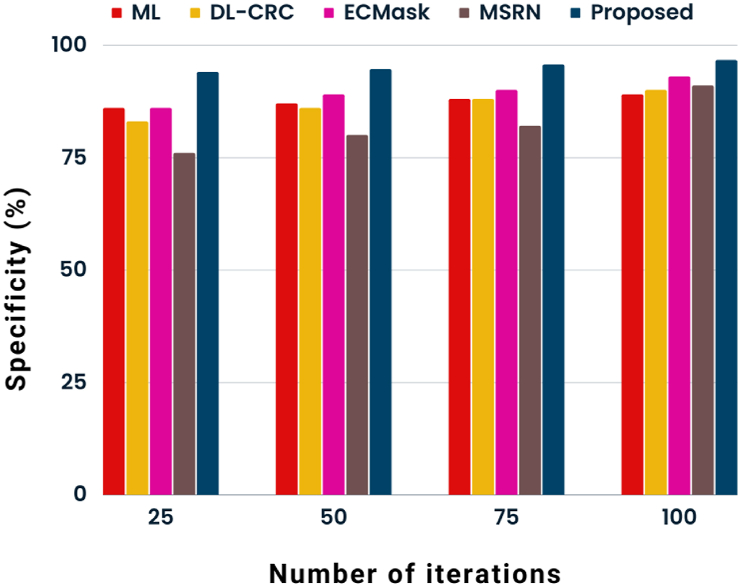
State-of-art analysis of specificity.

The graphical depiction of specificity rate analysis about various methodologies is shown in Fig. 5. The proposed POA-RCNN classifier is shown to outperform ML [9], DL-CRC [11], ECMask [14], and F-RCNN [15] approaches by comparing its specificity rate to that of existing methods. There were 25–100 iterations in all. The proposed POA-RCNN classifier has a specificity rate of 96.65 % at the 100th iteration, compared to specificity rates of 89 %, 90 %, 93 %, and 91 % for the ML, DL-CRC, ECMask, and F-RCNN algorithms.

## 5 Conclusion

In a nutshell, the proposed work is based on the smart monitoring system for monitoring COVID-19 patients. This system effortlessly monitors the patients remotely which is connected to the nearby city hospitals. The physicians monitor the health of the patients and avoid the spreading of the pandemic. For data capturing several smart sensors are used and designed in an IoT-based smart monitoring system. The data processing step is effectuated with the help of the proposed novel POA-based-RCNN approach which classifies the data as COVID-19 and normal. The performances are analyzed with some of the performance metrics such as accuracy, specificity, and sensitivity. The proposed system is compared with commercial sensors which are contactless and analyzed the effectiveness. The classification accuracy, sensitivity, and specificity of the proposed system are 97.12 %, 96.74 %, and 96.65 % respectively and thus surpass all the other approaches. The findings suggest that this technique may be utilized extensively in remote locations that are connected to hospitals in nearby urban centers. Our method provides a novel Recurrent Convolutional Neural Network (RCNN) based Puzzle optimization algorithm (PO) for classifying the data acquired by the monitoring system and stored in the cloud.

## Author contribution statement

M. M. Vijay: Liping Yu: Conceived and designed the experiments; Wrote the paper. J. Sunil: Conceived and designed the experiments; Contributed reagents, materials, analysis tools or data. V. G. Anisha Gnana Vincy: Muhammad Ijaz Khan: Performed the experiments; Contributed reagents, materials, analysis tools or data. V. Govindan: Performed the experiments; Analyzed and interpreted the data. Shahid Ali: Nissren Tamam: Barno Sayfutdinovna Abdullaeva: Analyzed and interpreted the data.

## Data availability statement

No data was used for the research described in the article.

## Declaration of competing interest

The authors declare that they have no known competing financial interests or personal relationships that could have appeared to influence the work reported in this paper.

## References

[1] Singh R.P., Javaid M., Haleem A., Vaishya R., Ali S. (2020). Internet of medical things (IoMT) for orthopaedic in COVID-19 pandemic: roles, challenges, and applications. Journal of Clinical Orthopaedics and Trauma.

[2] Wang C., Zhang F., Deng Y., Gao C., Li X., Wang Z. (2020). An adaptive population control framework for ACO-based community detection, Chaos. Solitons & Fractals.

[3] Deng W., Xu J., Zhao H., Song Y. (2020). A novel gate resource allocation method using improved PSO-based QEA. IEEE Trans. Intell. Transport. Syst..

[4] Karmore S., Bodhe R., Al-Turjman F., Kumar R.L., Pillai S.K. (2020). IoT-based humanoid software for identification and diagnosis of COVID-19 suspects. IEEE Sensor. J..

[5] Paranjape K., Schinkel M., Nanayakkara P. (2020). Short keynote paper: mainstreaming personalized healthcare--transforming healthcare through new era of artificial intelligence. IEEE Journal of Biomedical and Health Informatics.

[6] Vijay M.M., Punithavathani D.S. (2022). A memory-efficient adaptive optimal binary search tree architecture for IPV6 lookup address, in mobile computing and sustainable informatics. Proceedings of ICMCSI.

[7] Jiao H., Shen Q. (2020). Dynamics analysis and vaccination-based sliding mode control of a more generalized SEIR epidemic model. IEEE Access.

[8] Zaidan M.A., Motlagh N.H., Fung P.L., Lu D., Timonen H., Kuula J., Niemi J.V., Tarkoma S., Petäjä T., Kulmala M., Hussein T. (2020). Intelligent calibration and virtual sensing for integrated low-cost air quality sensors. IEEE Sensor. J..

[9] Loey M., Manogaran G., Taha M.H.N., Khalifa N.E.M. (2021). A hybrid deep transfer learning model with machine learning methods for face mask detection in the era of the COVID-19 pandemic. Measurement.

[10] Sakib S., Tazrin T., Fouda M.M., Fadlullah Z.M., Guizani M., Crc D.L. (2020). Deep learning-based chest radiograph classification for COVID-19 detection: a novel approach. IEEE Access.

[11] Awais M., Raza M., Singh N., Bashir K., Manzoor U., Islam S.U., Rodrigues J.J. (2020). LSTM-based emotion detection using physiological signals: IoT framework for healthcare and distance learning in COVID-19. IEEE Internet Things J..

[12] Abdulkareem K.H., Mohammed M.A., Salim A., Arif M., Geman O., Gupta D., Khanna A. (2021). Realizing an effective COVID-19 diagnosis system based on machine learning and IOT in smart hospital environment. IEEE Internet Things J..

[13] Otoom M., Otoum N., Alzubaidi M.A., Etoom Y., Banihani R. (2020). An IoT-based framework for early identification and monitoring of COVID-19 cases. Biomed. Signal Process Control.

[14] Kong X., Wang K., Wang S., Wang X., Jiang X., Guo Y., Shen G., Chen X., Ni Q. (2021). Real-time mask identification for COVID-19: an edge-computing-based deep learning framework. IEEE Internet Things J..

[15] Wang B., Zhao Y., Chen C.P. (2021). Hybrid transfer learning and broad learning system for wearing mask detection in the COVID-19 era. IEEE Trans. Instrum. Meas..

[16] Barnawi A., Chhikara P., Tekchandani R., Kumar N., Alzahrani B. (2021). Artificial intelligence-enabled internet of things-based system for COVID-19 screening using aerial thermal imaging. Future Generat. Comput. Syst..

[17] Zeidabadi F.A., Dehghani M. (2022). POA: puzzle optimization algorithm. International Journal of Intelligent Engineering & Systems.

[18] Wang D., He D. (2022). Fusion of mask RCNN and attention mechanism for instance segmentation of apples under complex background. Comput. Electron. Agric..

[19] Shanmugam T., Bansal S.A. (2023).

[20] Shahina E., Aravind P.K., Jarin T. (2020). THD reduction in execution of a nine level single phase inverter. International Conference on Communication and Signal Processing (ICCSP).

[21] F. Ndairou, M. Khalighi, L. Lahti, Fractional model of COVID-19 with pathogens as shedding effects. doi.org/10.48550/arXiv.2305.16689.

[22] Nabi K.N., Abboubakar H., Kumar P. (2020). Forecasting of COVID-19 pandemic: from integer derivatives to fractional derivatives, Chaos. Solitons & Fractals.

[23] Deng Q. (2020). Dynamics and development of the COVID-19 epidemic in the United States: a compartmental model enhanced with deep learning techniques. J. Med. Internet Res..

[24] Alali Y., Harrou F., Sun Y. (2022). A proficient approach to forecast COVID-19 spread via optimized dynamic machine learning models. Sci. Rep..

[25] Bhouri M.A., Costabal F.S., Wang H., Linka K., Peirlinck M., Kuhl E., Perdikaris P. (2021). COVID-19 dynamics across the US: a deep learning study of human mobility and social behavior. Comput. Methods Appl. Mech. Eng..

[26] Wu F., Liang X., Lei J. (2023). Modelling COVID-19 epidemic with confirmed cases-driven contact tracing quarantine. Infectious Disease Modelling.

[27] Kamalrathne T., Amaratunga D., Haigh R., Kodituwakku L. (2023). Need for effective detection and early warnings for epidemic and pandemic preparedness planning in the context of multi-hazards: lessons from the COVID-19 pandemic. Int. J. Disaster Risk Reduc..

[28] Liang X., Huang Z., Yang S., Qiu L. (2018). Device-free motion & trajectory detection via RFID. ACM Trans. Embed. Comput. Syst..

[29] Zeng Q., Bie B., Guo Q., Yuan Y., Han Q., Han X., Zhou X. (2020). Hyperpolarized Xe NMR signal advancement by metal-organic framework entrapment in aqueous solution. Proc. Natl. Acad. Sci. USA.

[30] Li Q., Lin H., Tan X., Du S. (2020). H∞ consensus for multiagent-based supply chain systems under switching topology and uncertain demands. IEEE Transactions on Systems, Man, and Cybernetics: Systems.

[31] Li Q., Miao Y., Zeng X., Tarimo C.S., Wu C., Wu J. (2020). Prevalence and factors for anxiety during the coronavirus disease 2019 (COVID-19) epidemic among the teachers in China. J. Affect. Disord..

[32] Jiang H., Xiao Z., Li Z., Xu J., Zeng F., Wang D. (2020). An energy-efficient framework for internet of things underlaying heterogeneous small cell networks. IEEE Trans. Mobile Comput..

[33] Song Y., Xin R., Chen P., Zhang R., Chen J., Zhao Z. (2023). Identifying performance anomalies in fluctuating cloud environments: a robust correlative-GNN-based explainable approach. Future Generat. Comput. Syst..

[34] Lu S., Ban Y., Zhang X., Yang B., Liu S., Yin L., Zheng W. (2020). Adaptive control of time delay teleoperation system with uncertain dynamics. Front. Neurorob..

[35] Gu Y., Chen Y., Wang X., Chen Z. (2023). Impact of COVID-19 epidemic on port operations: evidence from Asian ports. Case Studies on Transport Policy.

[36] Abbasi S., Sıcakyuz Ç., Erdebilli B. (2023). Designing the home healthcare supply chain during a health crisis. Journal of Engineering Research.

[37] Abbasi S., Daneshmand-Mehr M., Kanafi A.G. (2023). Green closed-loop supply chain network design during the coronavirus (COVID-19) pandemic: a case study in the Iranian automotive Industry. Environ. Monit. Assess..

[38] Abbasi S., Choukolaei H.A. (2023). A systematic review of green supply chain network design literature focusing on carbon policy. Decision Analytics Journal.

[39] Abbasi S., Daneshmand-Mehr M., Kanafi A.G. (2022). Designing sustainable recovery network of end-of-life product during the COVID-19 pandemic: a real and applied case study. Discrete Dynam Nat. Soc..

[40] Liao Q., Chai H., Han H., Zhang X., Wang X., Xia W., Ding Y. (2022). An integrated multi-task model for fake news detection. IEEE Trans. Knowl. Data Eng..

[41] Cheng B., Wang M., Zhao S., Zhai Z., Zhu D., Chen J. (2017). Situation-aware dynamic service coordination in an IoT environment. IEEE/ACM Trans. Netw..

[42] Zhuang Y., Chen S., Jiang N., Hu H. (2022). An effective WSSENet-based similarity retrieval method of large lung CT image databases. KSII Transactions on Internet & Information Systems.

[43] Zhuang Y., Jiang N., Xu Y., Xiangjie K., Kong X. (2020). Progressive distributed and parallel similarity retrieval of large CT image sequences in mobile telemedicine networks. Wireless Commun. Mobile Comput..

[44] Lu S., Ding Y., Liu M., Yin Z., Yin L., Zheng W. (2023). Multiscale feature extraction and fusion of image and text in VQA. Int. J. Comput. Intell. Syst..

[45] Lu S., Liu M., Yin L., Yin Z., Liu X., Zheng W., Kong X. (2023). The multi-modal fusion in visual question answering: a review of attention mechanisms. PeerJ Computer Science.

[46] Lv Z., Song H. (2019). Mobile internet of things under data physical fusion technology. IEEE Internet Things J..

[47] Abbasi S., Erdebilli B. (2023). Green closed-loop supply chain networks' response to various carbon policies during COVID-19. Sustainability.

[48] Abbasi S., Khalili H.A., Daneshmand-Mehr M., Hajiaghaei-Keshteli M. (2022). Performance measurement of the sustainable supply chain during the COVID-19 pandemic: a real-life case study. Found. Comput. Decis. Sci..

